# Clonality, outer-membrane proteins profile and efflux pump in KPC- producing *Enterobacter sp*. in Brazil

**DOI:** 10.1186/s12866-017-0970-1

**Published:** 2017-03-17

**Authors:** Juliana Ferraz Rosa, Camila Rizek, Ana Paula Marchi, Thais Guimaraes, Lourdes Miranda, Claudia Carrilho, Anna S Levin, Silvia F Costa

**Affiliations:** 1 Department of Infectious Diseases, University of São Paulo, Laboratory of Medical Investigation 54 (LIM-54), Hospital Das Clínicas FMUSP, São Paulo, Brazil; 2 Hospital de Itapecerica da Serra, Itapecerica da Serra, SP Brazil; 3 Hospital University of Londrina, Londrina, Paraná Brazil; 4 LIM-54, Faculdade de Medicina da Universidade de São Paulo, São Paulo, Brazil

**Keywords:** *E. aerogenes*, *E. cloacae*, Resistance, Carbapenems, Efflux Pump, Outer Membrane Proteins, β-lactamases and Activity efflux pump with inhibitor Carbonyl-cyanide-m-chlorophenylhydrazone (CCCP)

## Abstract

**Background:**

Carbapenems resistance in *Enterobacter* spp. has increased in the last decade, few studies, however, described the mechanisms of resistance in this bacterium. This study evaluated clonality and mechanisms of carbapenems resistance in clinical isolates of *Enterobacter* spp. identified in three hospitals in Brazil (Hospital A, B and C) over 7-year.

**Methods:**

Antibiotics sensitivity, pulsed-field gel electrophoresis (PFGE), PCR for carbapenemase and efflux pump genes were performed for all carbapenems-resistant isolates. Outer-membrane protein (OMP) was evaluated based on PFGE profile.

**Results:**

A total of 130 isolates of *Enterobacter* spp were analyzed, 44/105 (41, 9%) *E. aerogenes* and 8/25 (32,0%) *E. cloacae* were resistant to carbapenems. All isolates were susceptible to fosfomycin, polymyxin B and tigecycline. KPC was present in 88.6% of *E. aerogenes* and in all *E. cloacae* resistant to carbapenems. The carbapenems-resistant *E. aerogenes* identified in hospital A belonged to six clones, however, a predominant clone was identified in this hospital over the study period. There is a predominant clone in Hospital B and Hospital C as well. The mechanisms of resistance to carbapenems differ among subtypes. Most of the isolates co-harbored *bla*KPC, *bla*TEM and /or *bla*CTX associated with decreased or lost of 35–36KDa and or 39 KDa OMP. The efflux pump AcrAB-TolC gene was only identified in carbapenems-resistant *E. cloacae*.

**Conclusions:**

There was a predominant clone in each hospital suggesting that cross-transmission of carbapenems-resistant *Enterobacter* spp. was frequent. The isolates presented multiple mechanisms of resistance to carbapenems including OMP alteration.

## Background

Healthcare associated infections caused by *Enterobacter* spp. have increased in the last decade all over the world [1, 2]. Carbapenems are frequently used to treat serious infections caused by multi-resistant Gram-negative bacilli, especially those caused by over production of AmpC cephalosporinases or extended-spectrum β-lactamases (ESBL), such as infections caused by *Enterobacter* spp. Thus, the emergence of carbapenems resistance, defined as resistance to ertapenem, imipenem and/or meropenem, is becoming a therapeutic challenge [1, 2].

To date, carbapenemase is the most frequent mechanism of carbapenems resistance reported in *Enterobacter* spp [2, 3]. Studies have shown the presence of carbapenameses (*bla*KPC, *bla*IMP, *bla*VIM and *bla*NDM) in association with ESBL (*bla*TEM, *bla*SHV and *bla*CTX-M) in isolates of *E. aerogenes* and *E. cloacae* resistant to carbapenems [2, 3]. Although, *E. aerogenes* and *E. cloacae* carbapenems-resistant isolates can decrease and or loss OmpK 35–36 and 39KDa outer membrane proteins (OMPs), which lead to alteration of permeability and the induction of active drug efflux AcrAB-tolc, that contribute to resistance to carbapenems [2–6]. However, few studies demonstrated the importance of OMPs and efflux pump on carbapenems resistance in *Enterobacter* spp [1, 2, 7].

Therefore, the role of mechanisms of carbapenems resistance, such as OMP and efflux pump in *Enterobacter* spp, needs to be better addressed. The present study was conducted to investigate the clonality and mechanisms of carbapenems resistance in *E. aerogenes* and *E. cloacae* identified in three hospitals in Brazil.

## Methods

### Bacteria collection

One hundred and thirty *Enterobacter* spp. clinical isolates (105 *E. aerogenes* and 25 *E. cloacae*) identified in two hospitals (Hospital A and Hospital B whit 30 km of distance between them) in São Paulo in the state of São Paulo, and one hospital (Hospital C) 530 km distant, in Londrina in the state of Paraná, Brazil, were evaluated over a 7-year period, from 2005 to 2011.

Although, located in another state, the strains of *E. cloacae* identified in Hospital C in Londrina were evaluated in order to investigate the mechanism of carbapenems resistance and clonality in this species as well.

The identification of species was performed by API20 E (bioMérieux, France) and additional tests (Modify Rugai, Motility and Lysine).

### Clinical data

The following clinical and demographic data from the medical records of patients hospitalized in Hospital A and Hospital B, were registered: age, gender, underlying diseases, site of infection, length of stay in the Intensive Care Unit and death. Definitions (CDC) for the infections were those used by the Centers for Disease Control and Prevention. An Epi Info™ database was built, and results were expressed as means (standard deviation) or median (interquartile range), depending on normality. All data were analyzed anonymously and confidentially, with approval by the Research Ethics Committee of the three hospitals.

### Ethics statement

The study was performed in two hospitals located in São Paulo, Brazil, the Central Institute of Hospital das Clínicas of University of São Paulo (ICHC-FMUSP) and Hospital Itapecerica da Serra and one hospital in Paraná, the Universitary Hospital in Londrina. It was approved by the ethics committee of the hospitals. The approval number is 007/11.

### Susceptible testing

The minimal inhibitory concentrations (MICs) of imipenem (Merck & Co. Inc., Elkton, EUA), meropenem (Astra Zeneca), ertapenem (Sigma Chemical, St. Louis, Mo.), cefepime (Bristol-Myers Squibb, Guayaquil, Equador), polymyxin B (Sigma Chemical, St. Louis, Mo.) and tigecycline (Sigma Chemical, St. Louis, Mo.), was performed by broth microdilution with Mueller-Hinton broth. In addition, the MIC of fosfomycin (Sigma Chemical, St. Louis, Mo.) was performed using agar dilution as described in Clinical and Laboratory Standards Institute (CLSI). *P. aeruginosa* ATCC 27853, *E. coli* ATTC 25922, *S. aureus* ATCC 29213 and *E. faecalis* ATCC 29212, were used as control for all isolates (105 *E. aerogenes* and 25 *E. cloacae*). Carbapenems resistance was defined as: resistance to one or more carbepenems (ertapenem, imipenem and or meropenem) according with CLSI breakpoint.

### Carbapenemase genes and efflux pump

The presence of genes encoding ESBL (*bla*TEM, *bla*SHV, *bla*CTX-M), carbapenemases Class A (*bla*KPC, *bla*IMI and *bla*GES), Class B (*bla*IMP-1, *bla*VIM-2, *bla*GIM −1, *bla*SPM, *bla*NDM-1) and Class D (*bla*OXA-48), was investigated in all isolates (105 *E. aerogenes* and 25 *E. cloacae*) by PCR as described elsewhere (Table 1) [8–11], genbanks accession numbers: KF285575-KF285585, **KY524253 and MTZP00000000**.

**Table 1:** Primers of all carbapenems resistance genes studied and PCR annealing temperature

Primers	Sequences (5′-3′)	Annealing temperature	Size (pb)
*bla*TEM - F	TCGCCGCATACACTATTCTCAGAATGA	55	420
*blaTEM* - R	ACG CTC ACC GGC TCC AGA TTT AT		
*blaCTXM* - F	GCT CTAGAATTATTGCATCAGAAA CCGTG	55	893
*blaCTXM* - R	CGGAATTCATGATGACTCAGAGCATTGG		
*bla*_OSHV_ - F	TGCTTTGTTAATTCGGGCCAA	55	730
*bla*_SHV_ - R	ATGCGTTATATTCGCCTGTG		
*bla*_OXA48_ - F	GTAACAATGCTTGGTTCG	55	177
*bla*_OXA48_ - R	TGTTTTTGGTGGCATCGA		
*blakpc* - F	GTTACGCCAAAGGACGAAC		893
*blakpc* - R	TTTTCAGAGCCTTACTGCCC		
*bla*_SPM_ -F	CCTTTTCCGCGACCTTGATC	59	798
*bla*_SPM_ - R	ATGCGCTTCATTCACGCAC		
*bla*_SIM_ - F	GTACAAGGGATTCGGCATCG	58	569
*bla*_SIM_ - R	GTACAAGGGATTCGGCATCG		
*bla*_IMP_ -F	TTGGAAAATTATATAATCCC	47	188
*bla*_IMP_ - R	CCAAACCACTAGGTTATC		
*bla*_VIM_ - F	TTTGGTCGCATATCGCAAAG	60	382
*bla*_VIM_- R	CCATTCAGCCCAGATCGGCAT		
*bla*_NDM_ - F	GGCGGAATGGCTCATCACGA	60	375
*bla*_NDM_ - R	CGCAACACAGCCTGACTTTC		
AcrART - F	GAT TAT GAT TCT GCC TTG GCCG	60	130
AcrART - R	CAA TGC GAC CGC TGA TAG GGG		

PCR for all isolates (105 *E. aerogenes* and 25 *E. cloacae*) was performed to detect genes of efflux pump acrART using primers according to the study of Perez et al. [12].

DNA sequencing of genes of resistance was performed using the MegaBACE 1000, DNA Analysis System (Amersham Biosciences, UK. England), using DYEnamic ET Dye Terminator Kit (with Thermo Sequence™ DNA Polymerase II) US81090 code. The sequences were analyzed using the Sequence Analyzer software using the Cimarron Base Caller 3.12. The genetic sequence was compared with the database available on the Internet (BLAST - http://www.ncbi.nlm.nhi.gov/blast/).

### Pulsed-field gel electrophoresis

DNA analysis of all carbapenems-resistant isolates, 44 *E. aerogenes* and 8 *E. cloacae* were performed by PFGE, after digestion with XbaI Fast (Invitrogen) and the electrophoretic run was made with the following parameters: 23 h with pulse times ranging from 5 to 60s at 6 V/cm, using the CHEF-DR III System (Bio-Rad Laboratories, Richmond, CA, USA). DNA relatedness was computationally analyzed using BioNumerics v.7.1 software (Applied Maths, Sint-Martens-Latem, Belgium). The banding patterns were compared by using the unweighted pair-group method with arithmetic averages (UPGMA), with the Dice similarity coefficient required to be >80% for the pattern to be considered as belonging to the same PFGE type (dendrogram).

### Outer membrane protein profile

Based on clonality, the OMP of 22 *E. aerogenes* isolates and 5 *E. cloacae* isolates were analyzed. Bacterial outer membrane proteins (OMPs) were purified by treatment of the cell envelops with 2% sodium-N-lauryl sarcosinate (Sigma Chemical.St Louis., MO). The proteins were quantified by the Bradford method with the aid of Bradford reagent (Bio-Rad Laboratories, Brazil) by spectrophotometry at 595 nm (GeneQuant Pro, GE- Healthcare Life Sciences) and subsequently treated with beta-mercapto-ethanol at a ratio of 2 μl acid beta-mercaptoethanol acid to 10 μl protein. The proteins studied were applied to polyacrylamide gels prior manufactured 12.5% (GE Healthcare) at a concentration of 20 ng. Electrophoreses run was performed on the Multiphor II device (SG) at 600 V, 50 mA, and 30 W for about 60 min. The following molecular weights were used as markers: 97.4 KDa (phosphorylase B from rat muscle), 66,2KDa (bovine serum albumin), 45 kDa (egg albumin), 31,0KDa (bovine carbonic anhydrase), 21,51KDa (trypsin inhibitor) and 14,4KDa (lysozyme). *Enterobacter aerogenes* ATCC13048 was used as control. After electrophoresis, the gel was stained with Silver Staining Kit plus one Kit, Protein (GE Healthcare) according to the manufacturer’s instructions. We classified the protein profile based on intensity of band in without lack or loss of protein (++++), very little reduction of protein (+++), reduction (++), major reduction (+) and absent of protein based on previously described by Mostachio et al. [13].

### Efflux pump activity

The efflux pump activity was analyzed based on carbapenems’s MIC in 5 *E. cloacae* that harbored efflux pump gene and 5 in *E. aerogenes* belonged to different clones. MICs of imipenem, meropenem and ertapenem with 50 and 100 mg/mL and without the efflux pump inhibitors Carbonyl-cyanide-m-chlorophenylhydrazone (CCCP-Sigma Chemical.St Louis., MO) were determined by agar dilution to investigate the role of efflux pump on carbapenems-resistant *E. aerogenes* and *E. cloacae* isolates. The influence of an efflux pump on the carbapenems’s MIC for a given bacterial strain was determined by a reduction of at least four-fold of the respective MIC in the presence of CCCP [2].

## Results

A total of 44/105 (41, 9%) *E. aerogenes* and 8/25 (32, 0%) *E. cloacae* were resistant to carbapenems. Thirty-nine isolates of *E. aerogenes* resistant to carbapenems were identified in Hospital A, 5 isolates of *E. aerogenes* in Hospital B and 8 isolates of *E. cloacae* in Hospital C. *E. aerogenes* isolates presented MIC ranging from 2 to 128 mg /ml for imipenem, 4 to 64 mg /ml for meropenem and 1 to ≥128 mg /mL for ertapenem. *E. cloacae* isolates showed MIC ranging from 8 to 64 mg/ml for imipenem, 2 to 16 mg / ml for meropenem and 8 to 64 mg/mL for ertapenem. All isolates were susceptible to fosfomycin, polymyxin B and tigecycline (Table 2).

**Table 2:** In vitro activity of 7 antibiotics against 44 *E. aerogenes* and 8 *E. cloacae* carbapenems-resistant strains isolated in three Brazilians hospitals using microdilution and agar dilution

Antibiotics	Range	CIM 50 (mg/mL)	CIM 90 (mg/mL)	% resistance	CIM 50 (mg/mL)	CIM 90 (mg/mL)	% resistance
Imipenem	0,25–128	8	32	93,2	16	64	100
Meropenem	0,25–128	8	32	93,2	16	16	87,5
Ertapenem	0,25–128	64	128	86,4	64	64	100
Tigecycline	0,03–16	0,25	2	0	1	2	0
Fosfomycin^a^	0,25–256	16	64	0	16	32	0
Polymyxin B	0,003–16	1	2	0	1	2	0
Cefepime	0,25–128	≥128	≥128	100	≥128	≥128	100

^a^MIC by agar dilution

Demographic and clinical data of 39 patients with colonization and infection caused by *E. aerogenes* resistant to carbapenems in Hospital A and 5 in hospital B are shown in Table 2. Most of the isolates were from blood 12 (30.8%), followed by 9 (23%) from respiratory tract secretion and 6 (15.4%) from urine. Most of patients (*N* = 17) undergone surgery (4 liver and 2 kidney transplant), 2 patients received chemotherapy (1 acute leukemia and 1 bone marrow transplant) and 1 patient was HIV positive (Table 3). There was no clinical information regarding isolates identified in Hospital C, although, all isolates were from surveillance swabs.

**Table 3:** Clinical and demographic characteristics of 39 patients with infection and colonization caused by carbapenems-resistant *E. aerogenes* from Hospital A and 5 patients with colonization and infection caused by carbapenems-resistant *E. aerogenes* from Hospital B

Patients variables	Hospital A *N* = 39 (%)	Hospital B *N* = 5 (%)
Age ( range), mean	18–87 years old , 55.6 years old	24–88 years old, ,58.2 years old
Gender		
Female	13 (33,3%)	0
Male	22 (56,4%)	5 (100%)
Length of stay before identification of *E. aerogenes* (range), mean	6–190 days, 43.7 days	9–17 days, 16.2 days
Site of isolation		
Blood	12 (30,8%)	1 (20%)
Respiratory Tract secretion^a^	9 (23,1%)	0
Urine	6 (15,4%)	0
Peritoneal fluid	3 (8%)	0
Skin	2 (6%)	0
Others	7 (18%)	1 (20%)
Rectal swab	0	3 (60%)
Intensive care unit	23 (59%)	3 (60%)
Death	24 (61,5%)	1 (20%)

^a^Tracheal secretion and Bronco alveolar lavage (BAL)

The PCR showed that 39/44 (88.6%) *E. aerogenes* isolates harbored *bla*KPC, other genes encoding carbapenemases were not identified in any isolate. Thirty-nine (88.6%) had *bla*TEM-1 gene and 41 (93.2%) *bla*CTX-M gene. Among the eight isolates of *E. cloacae,* all harbored *bla*KPC and *bla*TEM-1 genes; other carbapenemases studied were not identified.

The outer membrane proteins of 22 of the 44 isolates of *E. aerogenes* and 5 of 8 isolates of *E. cloacae* were analyzed. The intensity of the proteins of interest Omp F (39 KDa) OMP C (42 KDa), 35–36KDa were compared with the molecular weight and ATCC *E. aerogenes* 13048 was used as control (Table 4).

**Table 4:** Minimum inhibitory concentration of carbapenems of 5 carbapenems-resistant *E. aerogenes* and 5 carbapenems-resistant *E. cloacae* isolates by agar dilution with or without efflux inhibitor CCCP

Antimicrobial agent	*E. aerogenes* (non-harboring acrART gene) *N* = 5						*E. cloacae* (harboring acrART gene) *N* = 5					
	50 mg/mL CCCP						100 mg/mL CCCP					
	MIC (mg/mL) SD						MIC (mg/mL)					SD
Imipenem	8	8	32	64	2	25,75	4	8	4	8	32	11,79
Imipenem + CCCP	≤0,125	≤0,125	≤0,125	≤0,125	0,25	0	≤0,125	≤0,125	≤0,125	≤0,125	≤0,125	0
Meropenem	4	4	8	32	8	11,79	4	4	2	8	16	5,58
Meropenem + CCCP	≤0,125	≤0,125	1	≤0,125	1	0	≤0,125	≤0,125	≤0,125	≤0,125	≤0,125	0
Ertapenem	32	64	32	256	32	93,38	16	16	8	32	32	10,73
Ertapenem + CCCP	≤0,125	2	≤0,125	2	4	1,61	8	8	≤0,125	≤0,125	≤0,125	4,35

All isolates harbored KPC

*SD* standart deviation

The protein profile was classified based on intensity of band in without lack or loss of protein (++++), very little reduction of protein (+++), reduction (++), great reduction (+) and absent (Table 4). All isolates resistant to carbapenems in this study showed a decrease in 35–36 KDa protein. We observed that the 42KDa protein in our analysis did not appear to be involved in carbapenems resistance.

*E. aerogenes* isolates with decrease of 39 KDa protein, presented CIM of 2–128 mg/mL to imipenem, 4–64 mg/mL to meropenem and 8 a ≥128 mg/mL to ertapenem. Isolates of *E. aerogenes*, which presented absence of 39 KDa protein showed MIC of 8 to 32 mg/ml for imipenem, 4 to 32 mg/ml for meropenem and 32–64 mg/mL to ertapenem.

*E. aerogenes* isolates with decrease of 35–36 KDa protein, presented MIC of 2–32 mg/ml for imipenem and 4 to 32 mg/ml for meropenem and 8–128 mg / mL to ertapenem. *E. aerogenes* isolates with absence of the protein of 35–36 KDa showed MIC of 128 mg/mL to imipenem, 64 mg/mL to meropenem and ≥128 mg/mL to ertapenem.

Moreover, all isolates of carbapenems-resistant *E. cloacae* showed decreased of 39KDa protein and absence of 35–36 KDa protein and their MICs to imipenem were 8 to 64 mg/ml, 2 to 16 mg/mL to meropenem and 8–64 mg/mL to ertapenem (Table 5).

**Table 5:** Outer membrane proteins, *bla*KPC gene, *bla*TEM and *bla*CTX and pump efflux (AcrART) profile of 22 carbapenems-resistant isolates of *E. aerogenes* (hospital A and B) and 5 *E. cloacae* (hospital C)

			Imipenem	Meropenem	Ertapenem	PCR				SDS-PAGE		
PFGE	Hospital	Date	MIC	MIC	MIC	*bla* KPC	*bla* CTX	*bla* TEM 1	acrART	42 KDa	39 Kda	35 e 36 KDa
A2	A	13/05/2005	32	8	32	+	+	+	-	++++	+++	++
A2	A	10/12/2005	16	8	64	+	+	+	-	++++	+++	++
C11	A	18/05/2006	32	16	128	+	+	+	-	++++	+++	++
C11	A	13/07/2006	32	16	128	+	+	+	-	++++	++++	++
C8	A	14/07/2006	16	16	64	+	+	+	-	++++	++++	++
C7	A	02/01/2007	16	4	32	+	+	+	-	++++	Absent	++
C6	A	23/03/2007	16	8	64	+	+	+	-	++++	+++	++
C13	A	25/04/2007	8	4	32	+	+	+	-	+++	Absent	++
C14	A	21/09/2007	8	4	64	+	+	+	-	++++	+++	++
G1	A	10/11/2008	32	16	64	+	**-**	**-**	-	+++	Absent	++
C1	A	06/01/2009	16	32	64	+	-	+	-	+++	Absent	++
C2	A	31/08/2009	16	4	16	+	+	+	-	+++	++	++
C4	A	03/11/2009	32	16	64	+	+	+	-	+++	Absent	++
C5	A	29/11/2009	16	4	32	+	+	+	-	+++	+	++
C3	A	05/12/2009	4	4	16	+	+	+	-	+++	++	++
B1	A	16/03/2010	16	8	64	+	+	+	-	+++	Absent	++
F1	A	08/06/2011	128	64	≥128	+	**-**	**+**	-	+++	++	Absent
D2	B	26/12/2010	2	8	32	+	+	+	-	++++	+	++
D1	B	05/01/2011	4	8	32	+	+	+	-	++++	+	++
D1	B	05/01/2011	4	8	32	+	+	+	-	++++	+	++
D3	B	06/01/2011	4	8	8	+	+	+	-	+++	+	++
A2	C	19/11/2012	8	2	8	+	-	+	+	+++	+	Absent
B1	C	-	8	8	64	+	-	+	+	+++	+	Absent
B1	C	-	8	8	64	+	-	+	+	+++	+	Absent
A3	C	-	64	16	64	+	-	+	+	+++	+	Absent
A5	C	-	16	16	64	+	-	+	+	+++	+	Absent

**Fig. 1:**
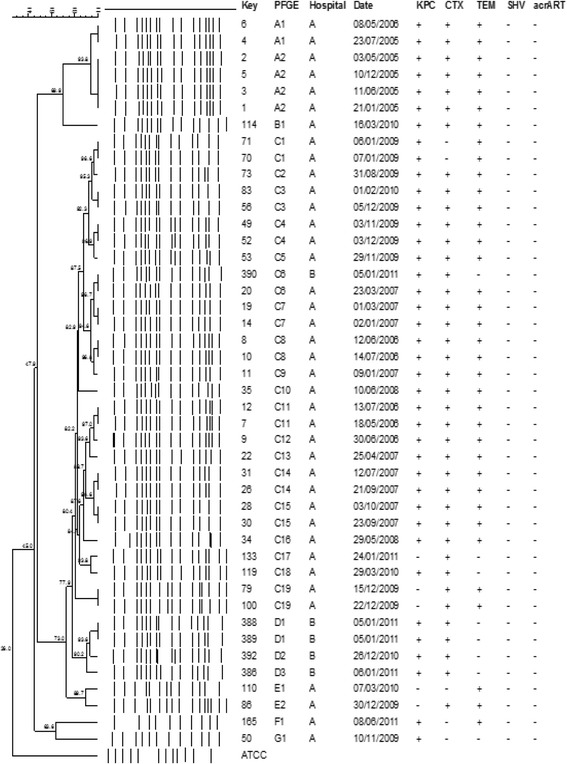
Dendogram of 39 carbapenems-resistant *E. aerogenes* isolates from Hospital A and 5 carbapenems-resistant *E aerogenes* from Hospital B

The PFGE showed 6 clones (A, B, C, E, F and G) among 39 isolates of carbapenems-resistant *E. aerogenes* identified in Hospital A. Clone A was present only in the first two year of study (2005 and 2006), and it was replaced by clone C that circulated during the entirely study. Twenty-seven (69.2%) isolates belonged to this predominant clone C that harbored KPC, TEM-1 and CTXM, whose subtypes ranging from C1 to C19 over the 7-year study period, from 2005 to 2011 (Fig. 1). Two subtypes (C17 and C19) of clone C and clone F were KPC negative.

The 4 carbapenems-resistant *E. aerogenes* identified in Hospital B belonged to a predominant clone nominated as clone D with subtypes from D1 to D3. This clone harbored KPC and TEM-1, and showed decreased of 39 and 35–36 KDa proteins (Fig. 1 and Table 5).

**Fig. 2:**
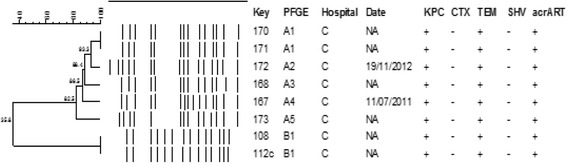
Dendrogram of 8 carbapenems-resistant *E. cloacae* isolates from Hospital C

In addition, the 8 carbapenems-resistant *E. cloacae* identified in Hospital C in Londrina belonged to two clones, clone A (predominant clone) and clone B (Fig. 2). All carbapenems-resistant *E. cloacae* isolates harbored KPC, TEM-1 and efflux pump gene acrART (Fig. 2). These clones showed as well lost of 35–36KDa proteins (Table 5).

The efflux pump AcrAB-TolC gene was only identified in carbapenems-resistant *E. cloacae*. All 5 isolates positive for this efflux pump were resistant to imipenem, meropenem and ertapenem with MICs above 8 mg/mL for all carbapenems (Tables 4 and 5).

The 5 carbapenems-resistant *E. aerogenes* isolates belonged to different clones and showed efflux pump activity in presence of CCCP inhibitor. Among the carbapenems-resistant *E. cloacae*, 2 of the 5 isolates showed efflux pump activity for meropenem, imipenem and ertapenem, and 3 isolates showed no activity on presence of ertapenem (Tables 4 and 5).

## Discussion

In the present study, we demonstrated that all *E. aerogenes* and *E. cloacae* isolates resistant to carbapenems were susceptible to polymyxin B, tigecycline and fosfomycin. KPC was the only carbapenemase identified. In contrast with previous studies [5, 10, 14], most of carbapenems-resistant *E. aerogenes* isolates co-harbored KPC and wide spectrum ESBL, such as *bla*TEM and blaCTX-M, in association with alteration of OMPs, mainly reduction or loss of 35–36 KDa and 39KDa proteins. In addition to alteration of OMPs, *E. cloacae* isolates harbored blaKPC; blaTEM and efflux pump gene acrART. *E. aerogenes* isolates were polyclonal, although a different predominant clone was finding in each hospital. On the other hand, *E. cloacae* belonged to two clones. The mechanisms of resistance to carbapenems differed among *E. aerogenes* subtypes. We identified isolates of *E. aerogenes* resistant to carbapenems that harbored only ESBL (TEM-1 and CTX-M) associated with decreased or loss of 35–36 kDa and 39 kDa proteins.

*E. aerogenes* and *E. cloacae* are important agents of healthcare-associated infections in several countries and resistance to carbapenems has been increasing in the last decade, thus there is a need for in vitro studies showing alternatives for the treatment of infections due to these microorganisms [3, 7, 14]. Since our susceptibility results showed that all carbapenems-resistant isolates were susceptible to tigecycline, fosfomycin, and polymyxin B, we consider these findings quite promising. However, it is noteworthy that despite the excellent results in vitro, pharmacokinetics and pharmacodynamics of tigecycline and fosfomycin, and the limited clinical experience in serious infections, especially bloodstream infections, are obstacles to the routine use of these antimicrobials in the treatment of systemic infections. Despite of these drawbacks, these drugs have been used with success as combination therapy in the treatment of severe infections caused by Enterobacteriaceae resistant to carbapenems [15, 16].

The demographic and clinical data from the studied hospitals showed that the most frequent site of isolation of carbapenems-resistant Enterobacter was blood, most of our patients had undergone surgical procedures, and had a high overall mortality, similar to previous studies [14, 17]. The only carbapenemase identified in our study was KPC. Data regarding carbapenems resistance in *Enterobacter* spp. in Brazil are scarce, although KPC is the carbapenemase most often described in *Enterobacter* spp. isolates in Brazilians hospitals [5, 7, 14].

Regarding the clonality*,* we identified in Hospital A, in São Paulo, 6 clones of *E. aerogenes*, named A, B, C, E, F and G over the study period, from 2005 to 2011, with the predominance of clone C. One predominant clone was also identified in hospital B in São Paulo. An intriguing finding is that one isolate of hospital B seems to be related to a clone of hospital A, pointed to a possible inter-hospital spread. Two clones A (subtypes A1 to A5) and B were identified among *E. cloacae* isolates resistant to carbapenems in Hospital C, in Londrina, Paraná. These findings suggest that cross-transmissions have occurred over the study period in all hospitals and highlight the importance of the stringent enforcement of hand hygiene and contact precautions to control the spread of this agent. Another interesting finding is that the mechanism of resistance to carbapenems in *E. aerogenes* from hospital A differs among subtypes and CTX-M was only identified in this hospital.

The profile of outer-membrane proteins of our isolates demonstrated that all isolates studied showed reduction of 35–36 KDa OMPs. These OMPs have been associated with β-lactam resistance in *Enterobacter* spp [18–21]. Studies in vivo and in vitro showed that diminished or loss of these OPMS were associated with increase on imipenem and meropenem MIC [18–22]. Other OMPs previously associated with carbapenems-resistance among *E. aerogenes* are OMPs of 42KDa and OMP of 39KDa [19]. However, in contrast with previous studies, we could not associate the alteration of the 42KDa OmpC with carbapenems resistance in our *E. aerogenes* and *E. cloacae* isolates. This finding could be explained perhaps by the clones that circulated in Brazil. Data regarding OMP in carbapenems–resistant Enterobacter in Brazil is limited. However, a previous Brazilian study demonstrated that OmpC and OmpF were present in only 6.6% of *E. cloacae* isolates resistant to ertapenem [5].

Few authors had evaluated the role of efflux pump on carbapenems resistance in *Enterobacter* spp [23, 24]. Efflux pump AcrAB system has been described as associated with resistance to meropenem and imipenem in *E. cloacae* [2, 22, 23]. In carbapenems-resistant *E. aerogenes* there were descriptions of efflux pump associated with KPC and/or porin loss as well [2, 23, 24]. In our study, the efflux pump AcrART was found only in *E. cloacae*. However, eventhough *E. aerogenes* isolates did not present the efflux pump AcrART gene, carbapenem efflux pump activity was observed in the presence of CCCP inhibitor. Possibly, these *E. aerogenes* isolates have other efflux pump, not described yet. These findings reinforce the importance of association of mechanism of resistance in carbapenems-resistant *Enterobacter* spp.

This study has several limitations: besides being retrospective, we evaluated only three hospitals and we could access the clinical data from only two hospitals. However, we were able to demonstrate that there was an association of mechanism of resistance such as β-lactamase, alteration of outer-membrane protein and efflux pump in carbapenems-resistant *Enterobacter* spp. in Brazil.

## Conclusion

In conclusion, we observed that there was a predominant clone in each hospital, suggesting that cross-transmission of carbapenems-resistant *Enterobacter* spp. was frequent. The isolates presented multiple mechanisms of resistance to carbapenems, such as KPC, ESBL and alteration of the outer membrane protein. *E. cloacae* presented as well the efflux pump AcrART, and in vitro activity of efflux pump inhibitor. These findings can be useful to control the spread of carbapenems resistance among *Enterobacter* spp. in the hospital setting.

## Abbreviations

ATCC: American Type Culture Collection
CCCP: Cyanide 3-chlorophenylhydrazone
DNA: Deoxyribonucleic acid
*E. aerogenes*: *Enterobacter aerogenes*
*E. cloacae*: *Enterobacter cloacae*
ESBL: Extended-spectrum β-lactamases
KPC: Klebsiella pneumonia carbapenemase
M-PCR: Multiplex PCR
NDM: New Deli metallo- β-lactamase
OMP: Outer-membrane protein
PCR: Polymerase Chain Reaction
PFGE: Pulsed-Field Gel Electrophoresis
VIM: Verona integrin metallo-β-lactamase
